# The role of vote advice application in direct-democratic opinion formation: an experiment from Switzerland

**DOI:** 10.1057/s41269-022-00264-5

**Published:** 2022-10-26

**Authors:** Isabelle Stadelmann-Steffen, Hannah Rajski, Sophie Ruprecht

**Affiliations:** 1 Institute of Political Science, University of Bern, Fabrikstrasse 8, 3012 Bern, Switzerland; 2 Graduate School of Economic and Social Sciences, University of Mannheim, Constance, Germany

**Keywords:** Vote advice application, Referendum, Vote, Vote intention, Opinion formation

## Abstract

In an increasingly digitized world, vote advice applications (VAAs) seem to be effective in providing voters with personalized information about their own positions vis-à-vis parties’ positions and specific policies. Even though electoral research has increasingly paid attention to the role VAAs play in voter’s opinion formation, very few studies have examined VAAs in the context of direct-democratic decisions. This article fills this gap by providing new insights into how VAAs affect individual decision-making in popular votes theoretically and empirically. We use novel data from the referendum campaign on the 2017 new energy law in Switzerland: a VAA experiment carried out in the framework of a three-wave panel survey. In the third wave, which took place a week before the referendum, respondents were randomly assigned to a treatment group and a control group; only the former was shown the VAA and made to use it. The results indicate two main takeaways. First, that using a VAA has a tangible effect inasmuch as the share of undecided voters is smaller among the treatment than among the control group. Second, VAA usage can have both a persuasive effect (i.e., it can change vote intentions) and an intensifying effect (i.e., it can strengthen voters’ preexisting intentions).

**Supplementary Information:**

The online version contains supplementary material available at 10.1057/s41269-022-00264-5.

## Introduction

According to Robert Dahl’s understanding of an “enlightened democracy,” people make political decisions in an informed way (see Mayer and Wassermair 2010, p. 173). However, public opinion research, in general, and research on voter decisions in direct democracy, in particular, have repeatedly argued that this is a very challenging goal, because in reality voters are neither capable nor willing to process all necessary information and, consequently, are most likely to make decisions based on low levels of knowledge or to rely on cues (Christin et al. 2002, p. 759; Colombo 2016; Colombo and Kriesi 2017; Neijens and De Vreese 2009).

We argue that vote advice applications (VAAs) can be an effective tool in our increasingly digitized context, because they provide voters with personalized information about their own positions vis-à-vis the positions of parties or policies. Like other decision aids (Neijens and De Vreese 2009; Neijens et al. 1992), VAAs do not only provide (new) information but also potentially help voters to more efficiently structure their opinions about an issue or a person. While research on the role VAAs play in voters’ opinion formation has increased over the years (Alvarez et al. 2014; Ladner 2012), it has almost exclusively focused on the electoral context, i.e., on situations when citizens vote for parties or candidates. In contrast, very few studies examine the role VAAs play in citizens’ opinion formation in direct-democratic decisions.

This study fills this gap by providing new insights into how VAAs affect individual decision-making in direct democracy both theoretically and empirically. More precisely, we ask *whether and how the use of VAAs affects individuals’ opinion formation in a referendum context*.

We draw on the literature on VAAs in the electoral context and on studies on individuals’ opinion formation in direct-democratic decisions to conceptualize a vote advice application as a “question and answer” tool. Users first answer questions about their stances on certain topics; the application then compares these stances to the positions of parties or candidates, or to specific issues (Alvarez et al. 2014; Ladner 2012; Sudulich et al. 2014). Ideally, the use of a VAA thus facilitates opinion formation and eventually even helps voters make decisions based on their “true” preferences.

The relevance of our study is at least twofold. First, it provides new insights into the role of VAAs in individuals’ opinion formation in the context of direct-democratic decisions. In contrast to elections, where most people have an initial idea about what party to vote for thanks to long-lasting party affiliations or some degree of closeness to a particular party, many voters enter direct-democratic campaigns in a state of relative ignorance (Converse 1964; Fishkin and Luskin 2005). This is reflected not least high shares of undecided voters at the beginning of a campaign (regarding the referendum under investigation in this study, see Dermont and Stadelmann-Steffen 2019). VAAs may therefore be even more important to voters’ opinion formation in such campaigns than they are in electoral contexts. All the more so because voting on ballot proposals may look like a simple yes/no decision but is the result of multidimensional choices. A specific ballot proposal consists of various elements; a voter may like some of those and reject others: she is faced with trade-offs. Decision making thus becomes a complex endeavor, whereby individuals require information to weigh the pros and cons of each proposal (Dermont and Stadelmann-Steffen 2019; Stadelmann-Steffen and Dermont 2018). The use of direct-democratic instruments is steadily growing around the world, which calls for additional research, especially as far as the mechanisms behind VAAs’ role in individuals’ voting decisions are concerned. Moreover, VAAs may also be attractive in contexts where direct-democratic instruments are used relatively rarely or have only recently been introduced, as they may compensate for the (still) lacking direct-democratic campaign structures in these environments (Heidbreder et al. 2019).

Second, we go beyond existing research by examining two potential mechanisms through which a VAA can influence voters’ opinion formation: The first relates to the mere *use* of a VAA, while the second refers to the *message* it conveys, i.e., the degree to which the user agrees with the policy at stake. Moreover, we differentiate between a *persuasive effect* (i.e., the idea that VAAs provide voters with new information or information that is different from what they would have at their disposal without the VAA), and an *intensifying effect* (i.e., the phenomenon of the VAA affirming voters’ original positions and therewith strengthening their vote intentions). It is worth noting that similar mechanisms are likely also at play during electoral campaigns (see Holbrook and McClurg 2005, p. 689, who refer to a similar mobilizing effect with respect to voter turnout), so our results may also be relevant to the role VAAs play in the electoral context.

We use novel data from the referendum campaign on the new energy law in Switzerland, which was collected in May 2017. Our analysis is based on a three-wave panel survey and a VAA experiment that was implemented in its last wave. In this third wave, which took place a week before the referendum, respondents were randomly assigned to a treatment and a control group. Only the former were shown the VAA and made to use it. A comparison of the treatment and the control groups provides evidence that the *use of a VAA* affects vote intention. Moreover, our analysis of respondents who had not yet voted by post (i.e., those respondents whose vote intentions could still be changed by their participation in the VAA) suggests that the *VAA’s message* could generate both a “persuasive effect” and an “intensifying effect,” depending on respondents’ original vote intentions and party affiliations.

## Theoretical background

### State of research

VAAs are designed to inform users about their optimal vote choice by supplying information about issues or party positions (Alvarez et al. 2014; Sudulich et al. 2014), thereby focusing on the outcome of decision-making (Price and Neijens 1998, p. 147f.). Existing VAAs and previous research on the topic have been almost exclusively limited to the context of elections, where VAAs “match” voters and parties. In so doing, VAAs usually compare voters’ and parties’ positions on numerous policy statements and indicate the degree of agreement between voters and parties. The selection of statements is pivotal, as the number of questions and their wording decide how well the different issues and their related aspects and positions are covered. Most VAAs also allow users to weight issues according to each issue’s perceived importance to the user. VAAs apply matching to calculate similarity scores (Mendez 2014) and existing research has demonstrated that, at least for salient issues, VAAs’ matching mechanisms work reasonably well (see Wagner and Ruusuvirta 2012).

The main questions surrounding VAAs thus come down to whether and how VAAs affect voters and their behavior. The optimistic account expects that VAAs guide users’ political opinions in independent and impartial ways (Holleman et al. 2016). The notion of proximity voting inherent to VAAs (Mendez 2014) is closely related to issue voting, which assumes that a voter’s proximity to the position of a party or an issue determines her vote choice (Garzia and Marschall 2019; Ladner 2012). The latter has become more relevant since the 1970s, when previously stable social cleavages started to lose importance. Furthermore, VAAs are theorized to strengthen the relationship between parties, citizens and (in some cases) the media, as a VAA positions itself squarely where these three players interact (Krouwel et al. 2014).

A more skeptical view, however, emphasizes that VAAs could foster unequal exclusion or “short-cut” decision-making. On the one hand, those unable to use the technology cannot benefit from VAAs; on the other hand, the voters who exclusively rely on VAAs for their decision-making reduce their democratic involvement to the last step of making a yes/no decision on an issue (Cedroni 2010). Furthermore, even though they bolster issue proximity, VAAs disregard other forms of representation, such as politicians’ and parties’ credibility and accountability (Wagner and Ruusuvirta 2012). More generally, existing research may have overestimated VAAs’ effects by failing to employ an experimental design and account for self-selection effects (Munzert and Ramirez-Ruiz 2021).

Empirically, a large number of studies focusing on different dependent variables, such as party preferences, party choice, and electoral participation, have been conducted in various countries and produced mixed results. While some have found VAAs to have no effects or only small effects (e.g., Enyedi 2016; Israel et al. 2016; Mahéo 2016; Marschall and Schmidt 2010; Ramonaite 2010; Walgrave et al. 2008), others have documented more meaningful and stronger relationships (Andreadis and Wall 2014; Christensen et al. 2021; Garzia et al. 2014; Garzia et al. 2017; Gemenis and Rosema 2014; Germann and Gemenis 2019; Kamoen et al. 2015; Kamoen et al. 2019; Ladner and Pianzola 2010; Munzert and Ramirez-Ruiz 2021; Pianzola et al. 2019; Ruusuvirta 2010; Van de Pol et al. 2019; Wall et al. 2014). In the 2007 Swiss elections, for example, VAA use had a self-reported influence on the vote choices of 66.5% (Fivaz und Nadig 2010) to 70% (Ladner 2012) of their users. In the Netherlands, more recent studies have shown that voters are more likely to choose a party when it has been recommended by a VAA (Kleinnijenhuis et al. 2019; Wall et al. 2014).

To the best of our knowledge, a single study has so far examined the influence of VAA use on a non-party vote, namely on voting in the Brexit referendum. According to this study, voters were more likely to vote to “leave the EU” if they received policy information indicating that they were indeed closer to the “leave” side of the referendum (Trechsel et al. 2017).

In the following, we delve deeper into the role VAAs play in individuals’ opinion formation in a direct-democratic decision by applying and further developing existing arguments and findings from VAA research focusing on the electoral context.

### Theoretical argument and hypotheses

Based on existing research, we conclude that a VAA may affect an individual’s opinion formation through *two different mechanisms*. First, we expect that the mere use of a VAA can make a difference. Obviously, this is the basic idea of a VAA, which has received empirical support, e.g., in Switzerland (Ladner 2012), but it is also in accordance with the general expectation that receiving more information affects people’s opinion (Luskin et al. 2002). We should thus observe differences between the vote intentions of voters who use a VAA and those who do not use it. Second, the specific message a VAA conveys can also be significant. In this case, we would expect to see varying reactions to the VAA among its users, contingent on what the VAA’s recommendation looks like and how it relates to a voter’s original vote intention.

Moreover, we further theorize potential VAA effects and argue that the latter can be both *persuasive* (i.e., VAAs can affect voting intentions by providing voters with new or additional information about their own positions) and *intensifying* (namely, VAAs can affirm voters’ original positions and further encourage them to vote for a specific party—or, in the context of direct democracy, to cast a “yes” or a “no” vote on a proposal). We suggest that differentiating between these two effects may be particularly important in a referendum vis-à-vis an electoral context because ballot decisions are inherently more directly related to (new) issues. Every ballot vote addresses a specific issue and question, on which some voters may have some prior knowledge and positions, while others may enter the referendum campaign in a state of relative ignorance. It is logical to expect that VAAs affect these two groups of voters differently.

Both the use and the message of a VAA can theoretically generate a persuasive and an intensifying effect. If the mere use of a VAA has a persuasive or an intensifying effect, we should observe that voters who have used a VAA find it easier to form a clear opinion on the ballot proposal. As a result, the share of undecided individuals should be lower in this group than in the group of voters who have not used the application. Furthermore, regardless of whether it recommends a “yes” or a “no” vote, the VAA can be expected to influence the final vote intentions of those who use it, either through its intensifying effect or through its persuasive effect.

Based on these considerations, our first two general hypotheses are:

#### **H1**

VAA use decreases the likelihood that a voter remains undecided about a ballot proposal (usage effect).

#### **H2**

The stronger a VAA message is, the more likely an individual is to follow it (message effect).

In an attempt to delve deeper into the effect of a VAA’s message—i.e., to differentiate between the aforementioned persuasive and intensifying effects—we consider that the role of the VAA is moderated by an individual’s original vote intention and whether the VAA recommendation is in accordance or in conflict with it and with party cues.^1^

We first focus on those *voters who do not have a clear original vote intention*. Studies have shown that the more surprising a VAA’s recommendation is, the higher the likelihood of the voter changing their vote choice is (Ladner et al. 2012; Vassil 2011). This is in accordance with findings from research using Choice Questionnaires, indicating that individuals without a clear prior opinion are most reactive to new information (Bütschi 2004, p. 317f.; van Knippenberg and Daamen 1996). Undecided voters are, by definition, either not yet sure how to vote or simply have not informed themselves. A “surprising” VAA result—one that provides some new information about their positions—might therefore be particularly influential (Neijens and De Vreese 2009; Zaller 1992). For these voters, the persuasive effect manifests in the VAA’s message replacing their prior state of being undecided. Indeed, existing research offers empirical evidence that initially undecided voters are especially likely to cast their votes in accordance with their VAA results (Ruusuvirta 2010). These findings also lend support to the view that as the relevance of cleavage voting shrinks, VAAs could become an important means of overcoming voters’ lack of party identification (Mahéo 2016) and predispositions. The corresponding hypothesis thus reads:

#### **H3**

The association between the VAA’s message and voters’ final vote intentions is stronger for initially undecided voters than it is for voters with an original vote intention (persuasive message effect).

Individuals with an original vote intention can have the VAA result either confirm or challenge their original vote intentions (Vassil 2011). We discuss both scenarios.

If the advice matches the voter’s original vote intention, the user should perceive this as a confirmation of their initial intention, which should, therefore, be intensified. These dynamics are in line with the theory of “motivated reasoning”: People have directional goals they want to achieve; therefore, they tend to seek information that confirms their preexisting opinions in an effort to avoid cognitive dissonance (see Festinger 1962). Not all studies have been able to identify such effects but many have, and they conclude that whenever VAA results match voters’ original vote intentions, voters are further inclined to vote accordingly (Neijens et al. 1992; Talukder et al. 2021; Wall et al. 2014). These dynamics played out in the study about the use of VAAs in the context of the Brexit referendum: Those who received information that confirmed their original vote intentions were more likely to act on these intentions (Trechsel et al. 2017).

Prior vote intentions are often linked to an individual’s party affiliation or party preference. In this context, recent research on direct-democratic decisions has examined how party heuristics and specific policy information interact (Colombo and Kriesi 2017; Dermont and Stadelmann-Steffen 2019). Theoretically, party and policy information can be conceptualized as two different modes of information processing. Whereas systematic processing entails that one thoroughly understands and evaluates all (policy) information available, heuristic processing requires much less cognitive effort or motivation. For example, one can simply rely on one’s party position to reach a decision. Additionally, two principles underpin this model: the “least effort principle” and the “sufficiency principle” (Chaiken and Ledgerwood 2012). The former suggests that individuals try to form their decisions as efficiently as possible, investing minimal effort, time, and cognitive resources in reaching conclusions. The “sufficiency principle,” however, states that individuals strive to enhance their judgmental confidence and therefore put a great deal of effort into thinking about an issue. Furthermore, heuristic and systematic information processing can interact to influence evaluations (Gawronski and Creighton 2013). In this context, VAA results can be seen as (easily available) policy information. If the individual consults a VAA, she gathers and evaluates more information about her personal attitudes. Doing so could refer to a more systematic dimension of information processing, as the VAA provides cognitive arguments. Similar to our reasoning about voters’ original vote intentions, their preferred parties’ positions on a direct-democratic ballot proposal matching the VAA’s output can also be expected to strengthen their decision to vote accordingly.

This discussion leads to the following hypothesis:

#### **H4a**

Congruence between the VAA’s message and respondents’ original vote intentions is associated with intensified original vote intentions (intensifying message effect).

#### **H4b**

Congruence between the VAA’s message and respondents’ preferred party’s position is associated with intensified original vote intentions (intensifying message effect).

The situation is more complicated if the VAA’s advice does not match voters’ original vote intention or the party cue. When this is the case, two reactions are possible: The users could either ignore the new advice or consider it and possibly change their vote choice. The former scenario is consistent with motivated reasoning and the idea that individuals tend to disregard conflicting information (see Kunda 1990) and avoid cognitive dissonance (see Festinger 1962). Similarly, individuals with strong prior attitudes are harder to influence (e.g., Neijens and De Vreese 2009; Zaller 1992). The latter relates to Dahl’s (1989) understanding that individuals are rational information processors who choose information based on accuracy goals and with the goal of reaching an enlightened decision. If individuals hold prior opinions about an issue, they use these opinions to “anchor” their evaluations of the new information available to them, which, if credible, is then used to update these prior opinions (Taber and Lodge 2006). When a conflict between the VAA’s information and voters’ prior vote intentions emerges, some individuals will eventually decide to change their vote choice, while others will retain their prior intentions. Hence, while both reactions suggest varying ways of handling VAA information, we assume that, on average, a VAA result that challenges a prior vote intention will reduce the likelihood of the voter eventually voting according to her original intention^2^:

#### **H5**

Incongruence between the VAA’s message and voters’ original vote intentions is associated with a higher likelihood of them changing said vote intentions (via-à-vis respondents who receive a congruent VAA recommendation) (persuasive message effect).

## Research design

### Case selection

Our case is a popular vote on a new energy law that took place in May 2017 in Switzerland. We argue that Switzerland in general and this vote in particular are suitable cases for our analysis for several reasons. First, Switzerland is home to the greatest number of direct-democratic decisions in the world (Altman 2010) and VAAs exist for all national and many subnational elections^3^ (but not for direct-democratic votes). This presents some analytical advantages. Even though VAAs are not available for direct-democratic votes, the idea of using VAAs is common and citizens are used to it. Moreover, carrying out the VAA experiment during a real direct-democratic campaign makes the VAA exercise in the survey even more realistic. Overall, this setting benefits both the internal and the external validity of the study. Second, a single national decision was taken in May 2017, which rarely happens in Switzerland. Campaigns on different issues interfere with one another if several proposals are on the ballot on the same day. In contrast, individuals’ decision-making on this specific issue can be analyzed in isolation, which allows us to rule out any interferences by parallel campaigns.

Overall, we argue that Switzerland in general and the ballot proposal on the new energy law in particular provide the ideal conditions for us to investigate our hypotheses as far as internal validity is concerned. However, we do acknowledge the need to be cautious when we generalize our findings to other contexts, votes, or issues. In particular, policy information and, thus, VAA information is, of course, specific to each ballot question. Hence, we cannot rule out that our results are case-specific—i.e., vote- or issue-specific—as well.

### Data

This analysis is based on a three-wave panel survey. The first wave collected 2891 responses, the second wave—1841, and the third wave—1253. Overall, we have full data on 1,181 respondents who participated in all three waves. The three waves were conducted 10 weeks, a month, and a week before the vote, respectively. The first wave took place in a pre-campaign setting. At the time of the second wave, the campaign had just started to get into its “hot” phase, obvious in the start of paid media (newspaper) advertisements. It also generally coincided with the distribution of the postal ballots to all citizens. The ballot boxes for elections and popular votes close on Sunday at 12 p.m., but most voters return their ballots by mail before Sunday. The last wave took place in the last week of the campaign when many people had already cast their votes or reached a decision.

We used Qualtrics to collect the sample from online panels and targeted people over the age of 18 living in Switzerland. The sample used language, age, gender, and cantonal (subnational) unit quotas to be representative. The survey was carried out in all three official languages of Switzerland: German, French, and Italian. Nevertheless, recruiting enough Italian speakers for the repeated cross sections proved challenging, and as a result, the samples were only representative of the French and German parts of Switzerland. The sample was fairly similar to the Swiss population in terms of age and gender. Nevertheless, like in most surveys—the groups with low education and low income were somewhat underrepresented (FSO 2019, 2020).

The VAA treatment was implemented in the third wave. Respondents were randomly assigned into a treatment group and a control group; the VAA was used by the treatment but not by the control group. As Table 4 in the Appendix demonstrates, the treatment and control groups did not significantly differ in terms of respondents’ sociodemographic composition and original vote intentions (see also Table S2 in the Supplemental Online Information (SI)). This uniformity suggests that randomization worked well.

The treatment group was presented with ten statements about the new energy law (for further details, see Table 5 in the Appendix). Respondents could use a slider to indicate positions ranging from 0 (clearly disagree) to 100 (clearly agree) (see Figure S1 in the SI). Moreover, respondents had to weight the importance of each statement—they had to indicate whether a statement was “not important,” “of average importance,” or “very important” to them. The VAA score was then calculated as a weighted sum of these answers: the degree of agreement with each statement $s$ was multiplied by its weight $w$ (either 50 (“not important”), 100 (“of average importance”) or 200 (“very important”)).^4^ This sum was then divided by the sum of the weights:

$$v= \frac{{\sum }_{i=1}^{10}{s}_{i}*{w}_{i}}{{\sum }_{i=1}^{10}{w}_{i}}$$

Respondents were then shown the individual percentage score of how much they agreed with the objectives of the law. The VAA score thus ranged from 0 to 100. Figure S3 in the SI depicts the distribution of the VAA scores. After receiving the VAA score, respondents were asked whether the result aligned with their expectations and whether it was relevant to them.

This setup implies that while the assignment to the VAA, its *use* in having respondents react to the ten statements, and their receiving their personal score make up an experimental setup, the treatment content, i.e., the *VAA’s message*, does not. In fact, the VAA recommendations shown to respondents naturally were realistic assessments of the latter’s positions based on the VAA items. Therefore, these assessments did not constitute a randomly defined information treatment.

Our main dependent variable is individuals’ vote intentions. We measured how respondents intended to vote in the referendum at the beginning of the survey in all three waves. In wave 3, we also asked whether they had already cast a vote (by postal voting). Moreover, an additional question at the end of the survey asked those respondents in the treatment group who had not yet voted by postal ballot about their vote intentions after receiving the information in the survey (namely, the VAA).^5^ The question in wave 1 included the answers “yes,” “no,” “undecided,” and “don’t know.” In waves 2 and 3, everyone except those who had already cast their votes (i.e., the early voters in wave 3) received a question about their likelihood of voting “yes” or “no.” To compare vote intention over time, values below 31% in waves 2 and 3 were coded as an intention to vote “no,” values between 31 and 74% as “undecided,” and scores equal to or greater than 75% were considered an intention to vote “yes.” We chose these asymmetric thresholds to account for the effects of social desirability bias—namely, in order to avoid counting too many respondents as “yes” voters if they were not (quite) certain that they would actually vote “yes.” This coding resulted in realistic groups as per the pre-ballot surveys and, eventually, the ballot result.

**Fig. 1:**
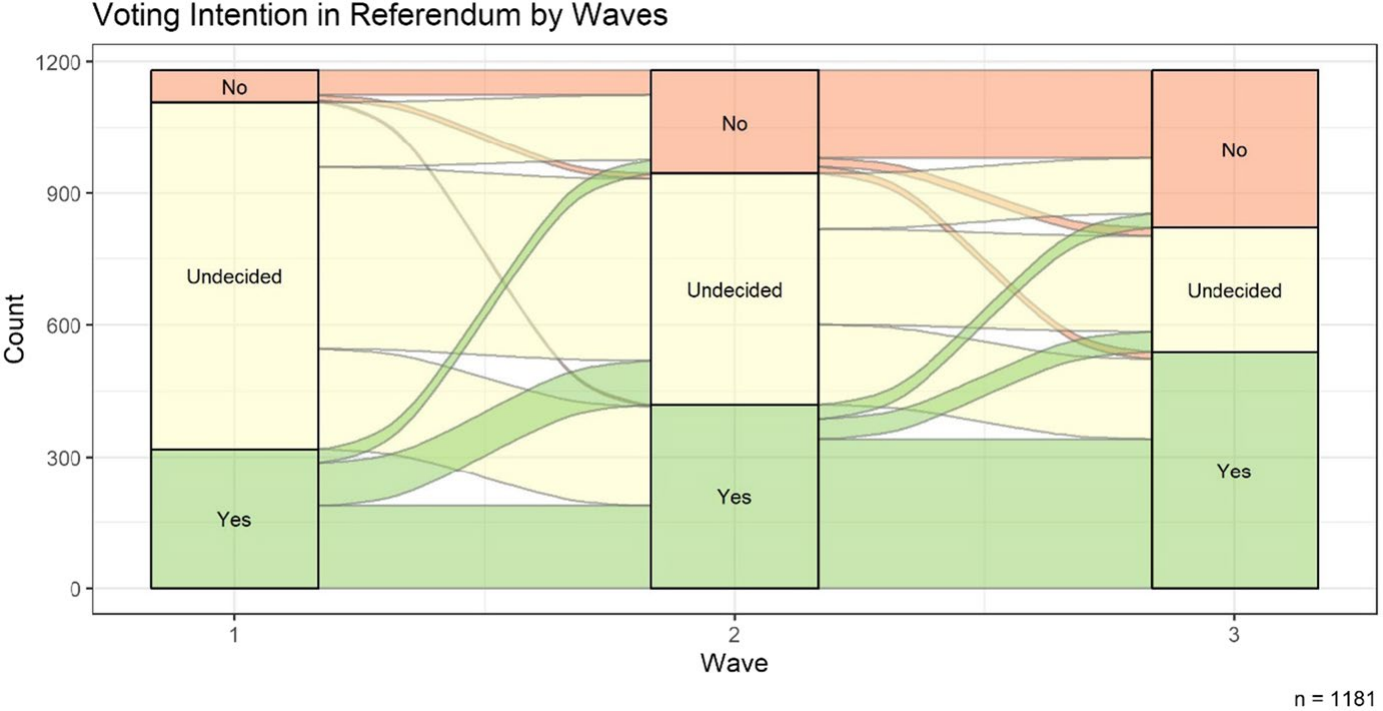
Vote intentions over the course of the campaign

Figure 1 shows voters’ mobility across the three waves, i.e., individuals’ vote intentions at different points in time, before any VAA treatment was set. The number of undecided voters decreased over time. At the same time, the proportions of those intending to vote in favor of and against the law, increased. Not unlike the actual referendum, in which 58.2% of the valid ballot papers were in favor of the law,^6^ a majority of the respondents indicated that they cast a “yes” vote. A comparison of the first and the third waves suggests that the campaign helped individuals form an opinion on the issue—the number of undecided voters greatly decreased.

The figure depicts respondents’ vote intentions in each of the three waves and mobility across the three waves for the sample (*n* = 1181) before any treatment was set.

We faced the problem that when the VAA was deployed in the third wave of the survey, 280 respondents in the treatment group and 268 respondents in the control group had already cast a vote by post. These members of the treatment group were not asked about their vote intentions after the treatment, because they had already voted. Moreover, we can assume that these voters might have been less prone to react to the VAA anyways, since they would have wanted to hold on to the vote they had already cast. For this reason, our analysis excludes these early voters. Hence, the results we present in the following pages are based on the 633 respondents who indicated their vote intentions in all three waves and had not yet cast their votes. Out of these individuals, 326 (including 53 who did not have the right to vote) saw the VAA and were asked about their post-treatment vote intentions.^7^

In the first empirical part, we rely on descriptive statistics and regression analysis to assess the effect of using a VAA (H1). We compared the treatment group’s vote intentions in wave 3 before and after using the VAA to the vote intentions of the control group in the same survey wave. Following H1, we hereby focus on whether the likelihood of being undecided differs between the treatment and the control groups.

In the second empirical part, we delve deeper into the mechanisms of the *VAA’s message* (H2-H5), concentrating on the treatment group, i.e., on those respondents who used the VAA. We employ OLS regression. We use the panel structure of the data and focus on within-individual variation in vote intentions—namely, on the change between a respondent’s vote intention in wave 1, which we consider her original vote intention, and her vote intention in wave 3 after she has used the VAA. We use respondents’ probabilities of casting a “yes” vote as a dependent variable and also account for their socio-demographics (education, age, sex, and household income), as well as their levels of political interest, their party identification, and party preferences (as measured in wave 1). To test the robustness of our results, we replicated the main models using respondents’ original vote intentions measured in waves 2 and 3, respectively, instead of those measured in the first wave. In fact, given that people’s vote intentions varied over the course of the campaign (especially among the initially undecided), the wave to which we choose to compare respondents’ final vote intentions may influence our results. At the same time, our theory does not inform us which comparison is most relevant to our study. Using the first wave, as we did in our main analyses, enables us to specifically investigate how initially undecided voters reacted to the VAA. Conversely, opting for wave 2 or 3 would probably allow us to distinguish the effect of the VAA from other campaign effects. We therefore present these models in the Supplemental Information (Tables S4 and S6).

More information about the variables, their operationalization, and their distributions can be found in Table 4 in the Appendix.

## Empirical results

### Testing the effect of using a VAA—comparing the vote intentions of the control and the treatment groups

Figure S4 and Table S2 in the SI document the self-reported voting intentions of the treatment and the control groups over the three waves. We observe that the two groups of voters were very similar in their vote intentions in waves 1 and 2. In the third wave, the treatment group was slightly more likely to oppose the ballot proposal than the control group (25% vs. 22%), before completing the VAA, however, this difference proved not to be statistically significant in an ordinal logistic regression. While the group of undecided voters was slightly larger in the control group, approximately 33% of the respondents in each group were in favor of the law. Presaging the actual ballot outcome, the “yes” voters formed the majority in both groups. Overall, these data suggest that the treatment and the control groups were similar before the treatment. We proceed to look at the effect of *using a VAA,* and more specifically at whether, as H1 posits, completing the VAA decreased one’s likelihood of being undecided.

Table 1 presents the results of the logistic regression models. The dependent variable is the likelihood that a respondent is undecided in wave 3. We use two slightly different measures. Models (1a) and (1b) use the vote intentions of the treatment and the control groups at the beginning of wave 3, i.e., *before the VAA treatment*. Those who indicated that they were undecided were coded 1 and all others are coded 0. We apply the same binary coding in Models (1c) and (1d), but this time we use the vote intentions of the treatment group *after the treatment*.

**Table 1:** The effects of VAA use on the likelihood of being undecided in wave 3

	Dependent variable			
	Undecided pretreatment		Undecided post-treatment	
	(1a)	(1b)	(1c)	(1d)
VAA treatment	− 0.132	0.185	− 0.158	0.323
	(0.160)	(0.315)	(0.161)	(0.312)
Undecided		0.962***		0.962***
		(0.267)		(0.267)
VAA treatment*undecided		− 0.444		− 0.669*
		(0.368)		(0.366)
Intercept	− 0.189*	− 0.879***	− 0.189*	− 0.879***
	(0.115)	(0.229)	(0.115)	(0.229)
Observations	633	633	633	633
Log likelihood	− 433.225	− 424.179	− 432.557	− 424.959
Akaike Inf. Crit	870.451	856.357	869.113	857.917

*Note* Log odds; standard errors in brackets

*=*p<0.1*, **=p<0.05, ***=p < 0.01

The main explanatory variable in all models is the use of the VAA—whether a respondent was in the treatment group and, thus, completed the VAA. Moreover, Models (1b) and (1c) control for respondents’ vote intentions in wave 1 and include an interaction between VAA use and vote intention in wave 1 to allow for the possibility that the effect of using a VAA is contingent on respondents’ original vote intentions. These models would confirm a significant treatment effect if there were a significant VAA effect in models (1c) and (1d) but not in models (1a) and (1b). We would interpret such results to mean that the likelihood of being undecided does not differ between the treatment and the control groups *before* the treatment, but a significant difference between the two does emerge *after* the treatment.

**Fig. 2:**
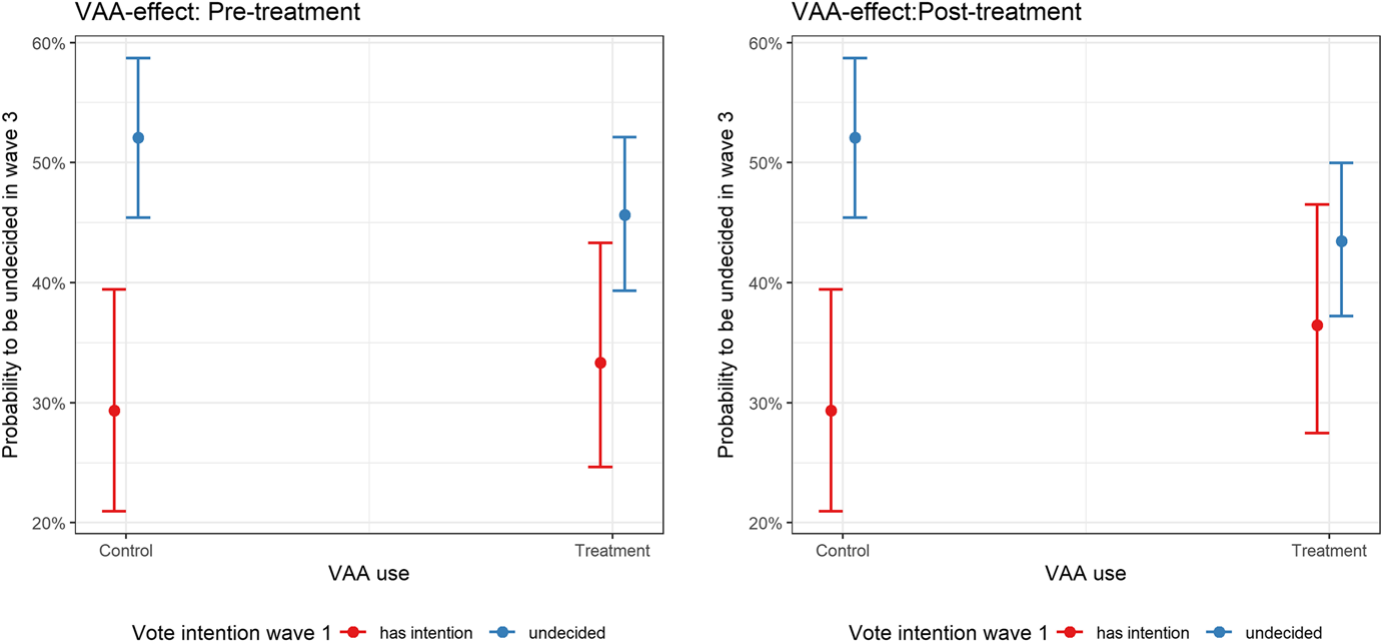
The effect of VAA use contingent on original vote intention. *Notes*: Predicted values based on Models (1b) (left panel) and (1d) (right panel)

Empirically, Models (1a) and (1c) do not lend support to such a pattern. The VAA variable is not statistically significant in either model. Models (1b) and (1d), however, reveal that when we account for whether individuals were undecided in wave 1, the effect of using the VAA (i.e., the treatment effect) differs significantly between respondents who were undecided in wave 1 and those who already had a vote intention at that early stage of the campaign. In fact, among the initially undecided, the likelihood of still being undecided shortly before the referendum was lower in the treatment group than in the control group. Conversely, individuals who had already indicated a vote intention in wave 1 exhibited a slightly higher likelihood of being undecided in wave 3 if they completed the VAA (however, here, the difference between the treatment and the control groups was not significant). The right panel of Fig. 2 illustrates the interaction effect using the post-treatment vote intention, while the left panel plots the interaction effect for the pre-treatment vote intention. The comparison of the two plots reveals that the overall patterns are similar in both cases. Thus, while the difference among the initially undecided is only significant after the treatment, which corroborates a VAA *usage* effect, the similar patterns imply that this treatment effect should not be overrated.

### Testing the effect of the VAA’s message within the treatment group

We proceed by delving deeper into the *VAA’s message mechanism*. The OLS estimations (Table 2, Models 1–3) initially lend support to H2: The more the VAA result bolsters a “yes” vote (or a “no” vote, respectively), the likelier an individual is to vote “yes” (or “no,” respectively). Hence, the VAA revealing that respondents’ issue-related attitudes are highly consistent with the ballot proposal is associated with a stronger post-treatment intention to vote “yes.” This pattern holds even when we control for original vote intentions.

**Table 2:** Linear regression models: probability of intending to vote “yes”

	(2)		(2a)		(3)		(4)		(5)		(6)		(7)	
Predictors	Estimates	*p*	Estimates	*p*	Estimates	*p*	Estimates	*p*	Estimates	*p*	Estimates	*p*	Estimates	*p*
Intercept	− 27.65	< 0.001	58.26	< 0.001	− 28.88	< 0.001	− 40.28	< 0.001	− 37.6	< 0.001	− 11	0.436	− 10.5	0.474
VAA outcome (range 0 to 100)	1.38***	< 0.001			1.37***	0.001	1.55***	< 0.001	1.47***	< 0.001	1.03***	< 0.001	1.02***	< 0.001
Voting intention: no (ref = undecided)			− 29.83***	0.001	4.99	0.458	13.86	0.381	17.31	0.276	19.85	0.277	21.28	0.248
Yes			13.57***	0.001	6.51**	0.031	51.32***	< 0.001	45.90***	0.001	47.14***	< 0.001	41.25***	0.002
VAA outcome x voting intention no							− 0.11	0.751	− 0.19	0.601	− 0.25	0.524	− 0.28	0.476
VAA outcome x voting intention yes							− 0.66***	**0.001**	− 0.60***	0.001	− 0.61***	0.002	− 0.54***	0.005
Favorite energy party: left (ref = none)									5.87	0.154	− 29.36*	0.091	− 29.08*	0.095
Center									6.03	0.15	− 33.60*	0.06	− 33.14*	0.067
Right									− 6.07	0.195	− 38.54**	0.036	− 41.74**	0.023
No answer									1.39	0.736	− 15.89	0.378	− 17.98	0.319
VAA outcome × left											0.56**	0.034	0.57**	0.031
VAA outcome × center											0.63**	0.021	0.61**	0.028
VAA outcome × right											0.54*	0.073	0.61**	0.042
VAA outcome × no answer											0.29	0.308	0.3	0.286
Control variables included	No		No		No		No		No		No		No	
Observations	326		326		326		326		326		326		326	
R^2^/R^2^ adjusted	0.486/0.485		0.077/0.071		0.494/0.489		0.513/0.505		0.527/0.514		0.538/0.519		0.566/0.533	

*Note:* Full results including control variables can be found in Table S3 of the SI, *=p<0.1, **=p<0.05, ***=p<0.01

The bold numbers indicated statistically significant results

As far as respondents’ original vote intentions are concerned, unsurprisingly, an original intention to vote “yes” (measured in wave 1; the reference category is “undecided”) is positively associated with a propensity to eventually cast a “yes” vote. In contrast, the coefficient of an original intention to vote “no” is no longer significant when the model controls for the VAA scores (from Model 3 onwards in Table 2). This might be because the number of intended “no” voters was very small in the first wave.^8^

Hypotheses 3, 4, and 5 go a step further by considering the interaction between respondents’ initial vote intentions and the specific message that the VAA conveys to those who use it. In this context, Figure S7 in the SI plots the degree to which respondents perceived the VAA result to be in line with their original vote intentions (Figures S5 and S6 in the SI compare the objective, rather than the subjective correlation between respondents’ VAA results and their original vote intentions, and confirm the following conclusions). According to Figure S7, a majority of respondents indicated that the VAA mostly confirmed their initial positions on the referendum proposal. It is unreasonable to assume that the VAA led these individuals to change their vote intentions (i.e., a persuasion effect); however, their certainty in their vote intentions could have increased (i.e., an intensifying effect, as suggested by H4).^9^

Conversely, the VAA provided new information to a substantial minority (roughly 30% of respondents)—i.e., it showed them that their prior vote intentions possibly did not reflect their issue-specific preferences. More specifically, 17.2% had expected a weaker agreement with the energy law and 12% a stronger one (7.7% did not know what to expect). This means that we can expect the VAA to have potentially changed this group’s final vote intentions, i.e., to generate a persuasive effect, either by pushing formerly undecided individuals to adopt a “yes” or a “no” intention (H3), or by triggering a change in their vote intentions from an original “yes” to a later “no,” and vice versa (H5).

While these descriptive results suggest that all three hypotheses have potential, we proceed to test their specific mechanisms. Model (4) in Table 2 corroborates that the VAA’s message effect is indeed contingent on whether an individual had an original vote intention.

**Fig. 3:**
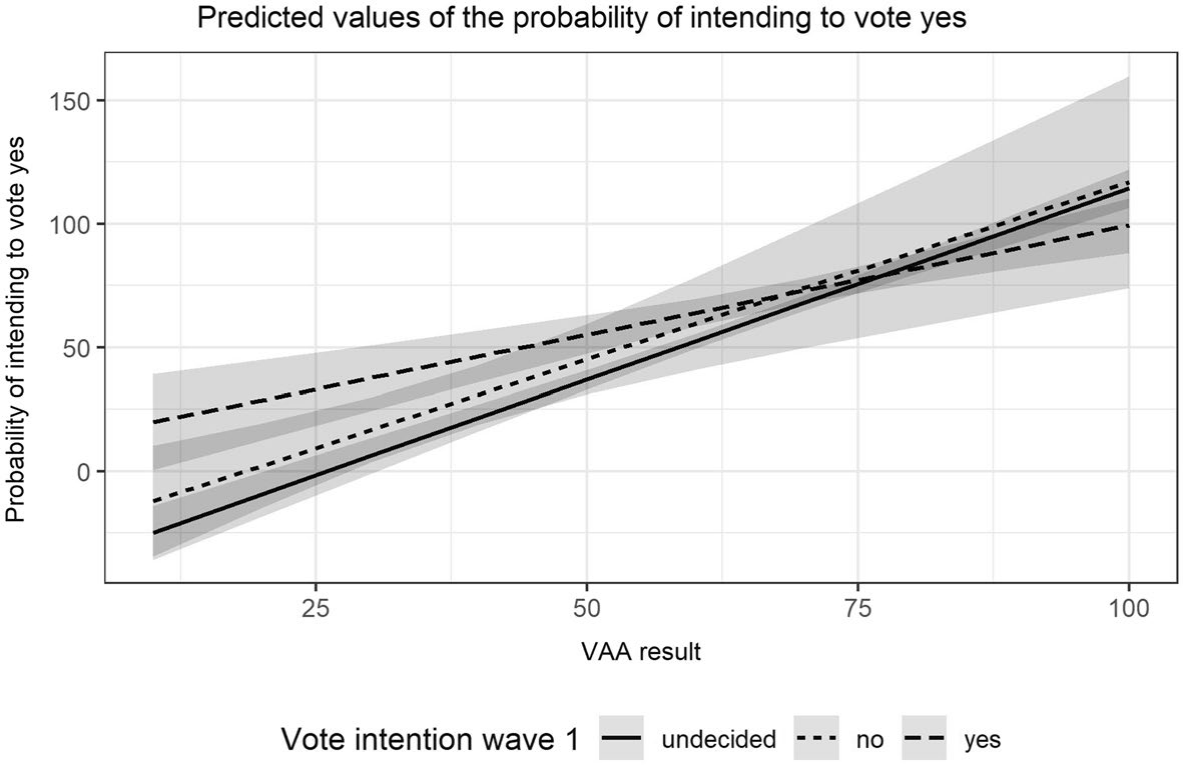
The VAA’s effect contingent on original vote intention. *Note:* Predicted probabilities calculated based on Model (4), Table 2

Figure 3 plots this interaction and lends (only) partial support to Hypothesis 3—that originally undecided voters are most strongly affected by the message of the VAA. The slope is steeper for undecided individuals than it is for “yes” voters, but the relationship between respondents’ VAA results and their final vote intentions is not significantly weaker for those who had originally intended to vote “no” (for a similar finding and the use of an information and choice questionnaire, see Neijens and De Vreese 2009)

.^10^

These findings also provide a first indication that the VAA’s message can have both an intensifying effect (namely, a high VAA result can solidify an original intention to vote “yes”) and a persuasive effect (i.e., it can provide some new (potentially contrasting) information, as H4 and H5 suggest). More explicitly, we test the validity of H4 and H5 in two steps. First, we follow H4 and look at the role of party affiliation (Models (6) and (7) in Table 2). Then, we delve deeper into the role of respondents’ original vote intentions (Table 2).

As far as party affiliation is concerned, Models (6) and (7) in Table 3 show that party affiliation and its interaction with respondents’ VAA results are indeed associated with final vote intentions. Overall, respondents who favor right-wing parties were likelier to reject the ballot proposal. Importantly, however, we observe positive interaction terms between respondents’ VAA scores and all party groups. Respondents who adhered to a party had a stronger reaction to the VAA’s message than those who did not have or indicate a party preference. A high VAA result thus greatly increased the likelihood of a respondent eventually voting “yes” on the proposal across the entire political spectrum (from left to the right).^11^

All presented results are robust to the inclusion of additional sociodemographic and socioeconomic variables (Model (7)). Moreover, these variables increase the explained variance in the probability of a final intention to vote “yes” (with an adjusted R^2^ of 0.531).

To gain more insight into the interaction between respondents’ VAA results and their original vote intentions, we create a new variable that more specifically captures these two features. The VAA score was coded as: 0-30-“no,” 31-74-“undecided,” and 75-100-“yes.”^12^ These VAA scores were then cross-tabled with respondents’ initial vote intentions, which resulted in a variable with nine possible outcomes: “no (vote intention) & no (VAA result),” “no & undecided,” “no & yes,” “undecided & no,” “undecided & undecided,” “undecided & yes,” “yes & no,” “yes & undecided,” and “yes & yes.” All outcomes could be observed in our sample, except the “yes & no” combination (an original intention to vote “yes” and a VAA recommendation to reject the proposal). The probability of a respondent intending to vote “yes” after receiving the treatment continues being our dependent variable.

Table 3 presents these regression results and confirms that the association between the VAA’s recommendation—its message—and respondents’ post-treatment vote intentions is significant. The more fine-grained analysis reveals that, depending on its result, the VAA can lead users’ opinions to move in both directions (toward both a “yes” and a “no” vote). As the results above and H3 would lead us to expect, we observe that the correlation between the VAA’s message and originally undecided voters’ final vote intentions is rather strong. Compared to undecided voters with an undecided VAA result, a VAA score below 31 is associated with a significantly and substantially lower probability to eventually vote “yes,” whereas a VAA outcome between 75 and 100 goes hand in hand with a significantly and substantially higher likelihood of accepting the ballot proposal. These results also hold when we control for age, sex, household income, and education. Hence, the findings confirm that the aforementioned aggregate trend toward a “yes” vote did not stem from VAA-induced biased opinion changes, but from the fact that many (undecided) individuals exhibited attitudes that came close to a “yes” vote and which were made visible to them by the VAA.

**Table 3:** Linear regression models —original vote intentions and VAA outcomes combined

	(8)		(9)	
Predictors	Estimates	*p*	Estimates	*p*
Intercept	48.22	< 0.001	44.52	< 0.001
Voting intention (w1) vs. VAA (ref = undecided & undecided)				
No & no	− 48.22***	< 0.001	− 46.41***	< 0.001
No & undecided	− 9.85	0.287	− 7.72	0.408
No & yes	42.78	0.095	40.4	0.116
Undecided & no	− 43.55***	0.004	− 45.96***	0.002
Undecided & yes	41.36***	< 0.001	41.58***	< 0.001
Yes & undecided	12.82***	0.002	11.88***	0.004
Yes & yes	41.36***	< 0.001	41.62***	< 0.001
Control variables included	No		Yes	
Observations	326		326	
R^2^/R^2^ adjusted	0.393/0.380		0.426/0.394	

*Note:* Full results including control variables are available in Table S5 of the SI,  *=p<0.10, **=p<0.05, ***=p<0.01

The bold numbers indicated statistically significant results

In a similar vein, the estimation results identify an intensifying effect for those respondents with congruent original vote intentions and VAA results. If a respondent intended to vote “no” and the VAA presented a similar result (i.e., < 31), the latter reinforced the former. Conversely, based on the model, when an original intention to vote “yes” is combined with a VAA score above 74, the probability of the voter declaring an intention to vote “yes” increases by more than 41 percent vis-à-vis the reference category. This lends support to H4.

Finally, our data do not allow us to provide systematic empirical support to the hypothesis that using a VAA leads to changes in respondents’ vote intentions, i.e., from an original intention to vote “no” to a final “yes” vote, or vice versa (H5). While no original “yes” voter received a really low VAA result, there was a small group of original “no” voters with moderate-to-high VAA results. However, group sizes are too small for us to identify significant differences.

Our robustness tests—when we use respondents’ original vote intentions from waves 2 and 3 instead of wave 1 (see SI, Tables S4 and S6)—largely confirm our results. Across all these models, the coefficient of the VAA’s message becomes somewhat smaller if we use respondents’ vote intentions at the later stages of the campaign, rather than in wave 1. This suggests that some changes in vote intentions stemmed from campaign effects, which might be related to the VAA outcome. For example, during the campaign, a previously undecided but right-leaning voter may be exposed to campaign information that pulls her in the direction of a “no” vote (i.e., provides the same directional information as a low VAA score). It is important to note that the VAA variable remains significant across all of these models. Hence, the outcome of the VAA remained a significant predictor of respondents’ final vote intentions even when we controlled for and considered voters’ vote intentions in the middle and at the end of the campaign.

## Conclusions

While previous research on VAAs has almost exclusively focused on electoral decisions, this paper joins the very few studies examining the role of VAAs in direct-democratic votes. We have used data from a three-wave survey on the 2017 referendum on the new energy law in Switzerland to investigate how using a VAA and receiving its message affected respondents’ final vote intentions. The most important findings can be summarized as follows.

First, our analysis lends some empirical support for a VAA *usage* effect: However, while we do not observe a general treatment effect, we find that for the subgroup of initially undecided, the likelihood of still being undecided shortly before the referendum is lower in the treatment group than in the control group.

Second, our analyses quite consistently point to a VAA *message* effect. Most generally, when respondents see their VAA results, they tend to declare a final vote intention strongly aligned with these results. This pattern holds across different groups—namely original “no” voters, original “yes” voters, and formerly undecided respondents. The results provide consistent empirical support for the idea that a VAA can have both an *intensifying effect* (i.e., one that assures voters with original vote intentions that they have “gotten it right” and, thus, reinforces their intentions) and a persuasive *effect* mainly on formerly undecided voters (i.e., one that enables these respondents to form a vote intention by virtue of the VAA providing them with new or additional information).

Third, the main implication of these findings is that VAAs have the potential to facilitate voters’ opinion formation in direct-democratic votes. A large share of voters enters a direct-democratic campaign in a state of relative ignorance. This share reflects the large proportion of undecided voters at the beginning of the campaign. Our results demonstrate that these individuals can use the information from the VAA to reach a decision on the ballot proposal, which may, eventually, also increase the likelihood that these individuals participate in the vote. Similarly, like party cues, VAAs may help reduce the uncertainty even of voters with prior vote intentions by reassuring them that they have made the right choice on an often complex ballot proposal.

Our study is not without limitations. First, we have analyzed vote intentions, rather than actual (reported) voting decisions. Nevertheless, we assume that our setup constitutes a very conservative setting to test for VAA effects, because VAA information may be particularly relevant to last-minute decisions by as-of-yet undecided voters. Second, we have focused on a single referendum in a single country (and have thus analyzed one particular VAA on a specific topic). Therefore, we cannot rule out the possibility that the results are driven by our chosen case. In particular, while on aggregate, our VAA users shifted toward a “yes” vote, we have no way of knowing whether the latter resulted from our VAA instrument or whether policy information increased popular support for ballot proposals on environmental and energy policy (see Dermont & Stadelmann-Steffen 2019). Hence, we could confirm that our VAA facilitated users’ opinion formation, as suggested by the VAA literature (Alvarez et al. et al. 2014; Sudulich et al. 2014), but we would not go as far as saying that it made voters find their “true” or optimal vote choice. Further research should therefore analyze the use of VAAs across different ballot decisions and topics. A third limitation has to do with the placement of the VAA shortly before the actual vote. While we sought to present the VAA to respondents at a stage when they had already reflected on the issue on their own, the disadvantage of our timing was that there were many early voters for whom we could no longer observe a change in intentions as a result of the VAA. Even when these changes were observed, they were short term, and we could not be sure that the VAA’s effect would persist over a longer period of time (i.e., for days or weeks). Fourth, our study also revealed some more general challenges related to the study of VAAs. The VAA results of a majority of respondents agreed with their original vote intentions. While this fact might be interpreted as a validation of the VAA instrument, it also means that for many respondents, a VAA could not conceptually lead to a behavioral *change* (i.e., a persuasive message effect for voters with original vote intentions). Empirically, this singles out a small number of cases in which VAA studies might be most interesting—namely where the VAA result diverges from an individual’s original vote intention and/or preferred party position, but where—as in our study—a lack of data does not allow for further investigation.

Nevertheless, we argue that our study is relevant despite these limitations and beyond our specific case. Most important, it documents, both theoretically and empirically, the need and relevance of investigating the role of VAAs in a direct-democratic context. We show that, compared to an electoral setting, it is particularly important to differentiate between VAA use and VAA message but also between a persuasive and an intensifying effect. More substantively, our results specifically point to the important interaction between original vote intention (or the absence of it), party cues, and the VAA. Future research should theoretically distinguish group-specific VAA effects and also collect larger comparative samples that allow for more fine-grained empirical analyses that consider context-specific variation.

### Electronic supplementary material

Below is the link to the electronic supplementary material.

Supplementary file1 (DOCX 1020 kb)

## Appendix

See Tables 4 and 5.

**Table 4:** Variables and descriptive statistics per group

Variable	Question	Descriptive statistics per group			
		Treatment group (*n* = 606)	Control group (*n* = 575)	Non-postal voters in treatment group (*n* = 326)	Population (2016, all ages)
Vote intention	*vox1.dec {forced response}* How do you think you will vote on the amendment to the Energy Act (EnG) (Energy Strategy 2050)? *vox1.decc (for noncitizens) {forced response}* How would you vote on the amendment to the Energy Act (EnG) (Energy Strategy 2050) if you were eligible to vote and participated? Response categories in wave 1: o Yes (1) o I have not decided yet (3) o No (0) o Don’t know / No response (98) Responses in waves 2 and 3: Likelihood to vote yes, 0–100% Recoded: Probability of voting “yes” < 31: “No” Probability of voting “yes” 31–74%: “Undecided” Probability of voting “yes” 75% and higher: “Yes”	*Wave 1* No = 39 (6.44%) Yes = 167 (27.56%) Undecided = 400 (66%) *Wave 2* No = 123 (20.3%) Yes = 224 (36.96%) Undecided = 259 (42.74%) *Wave 3— pretreatment* No = 189 (31.19%) Yes = 277 (45.71%) Undecided = 140 (23.1%) *Wave 3—pretreatment (metric)* Mean = 31 SD = 37 Min = 0 Max = 100	*Wave 1* No = 34 (5.91%) Yes = 151 (26.26%) Undecided = 390 (67.83%) *Wave 2* No = 114 (19.83%) Yes = 195 (33.91%) Undecided = 266 (46.26%) *Wave 3* No = 171 (29.74%) Yes = 262 (45.57%) Undecided = 142 (24.7%) *Vote intention (metric, overall)* Mean = 31 SD = 36 Min = 0 Max = 100	*Wave 1*No = 14 (4.29%) Yes = 82 (25.15%) Undecided = 230 (70.55%) *Wave 2*No = 56 (17.18%) Yes = 99 (30.37%) Undecided = 171 (52.45%) *Wave 3—pretreatment* No = 83 (25.46%) Yes = 106 (32.52%) Undecided = 137 (42.02%) *Vote intention (metric, overall)* Mean = 57 SD = 32 Min = 0 Max = 100 *Wave 3—post-treatment* No = 66 (20.25%) Yes = 125 (38.34%) Undecided = 135 (41.41%) *wave 3*—*post-treatment (metric)* Mean = 60 SD = 32 Min = 0 Max = 100	
Treatment	Has the individual received the treatment (VAA)?	Treatment: 100%	Control: 100%	Treatment: 100%	
VAA score	*vaaresult* Weighted mean of the ten VAA items, which can take values between 0 and 100. High VAA values mean that a respondent is close to a yes-position (in favor of the new law); low values indicate proximity to a no-position on the law	Mean = 64.07 SD = 17.73 Min = 10 Max = 100		Mean = 63.98 SD = 16.4 Min = 10 Max = 100	
Party	*partyenvir {randomized order of answers} {force response}* Which party's goals and demands correspond **most closely** to your own preferences in **energy policy**? Categorized: None: "none" Left: "SP", "GPS", "PdA" Center: "CVP", "GLP", "CSP", "EVP", "BDP", "FDP" Right: "SVP", "Lega dei Ticinesi", "MCG"" NA: NA, others	Left = 164 (27.06%) Center = 154 (25.41%) Right = 95 (15.68%) None: 86 (14.19%) NA = 107 (17.66%)	Left = 159 (27.65%) Center = 158 (27.48%) Right = 83 (14.43%) None = 91 (15.83%) NA = 84 (14.61%)	Left = 82 (25.15%) Center = 73 (22.39%) Right = 45 (13.8%) None = 51 (15.64%) NA = 75 (23.01%)	
Age	*agecat {force response}* What is your age?	18–24 = 53 (8.75%) 25–44 = 228 (37.62%) 45–64 = 208 (34.32%) 65 + = 117 (19.31%)	18–24 = 44 (7.65%) 25–44 = 205 (35.65%) 45–64 = 199 (34.61%) 65 + = 127 (22.09%)	18–24 = 27 (8.28%) 25–44 = 143 (43.87%) 45–64 = 106 (32.52%) 65 + = 50 (15.34%)	18–24 = 9.99% 25–44 = 28.61% 45–64 = 35.29% 65 + = 26.11%
Sex	*sex {force response}* What is your gender?	Female = 287 (47.36%) Male = 319 (52.64%)	Female = 254 (44.17%) Male = 321 (55.83%)	Female = 168 (51.53%) Male = 158 (48.47%)	Female = 50.44% Male = 49.56%
Education	*educ {force response}* What is the highest education you have completed with a certificate or diploma?	Secondary 1 = 29 (4.79%) Secondary 2 = 325 (53.63%) Tertiary = 241 (39.77%) NA = 11 (1.82%)	Secondary 1 = 31 (5.39%) Secondary 2 = 317 (55.13%) Tertiary = 223 (38.78%) NA = 4 (0.70%)	Secondary 1 = 17 (5.21%) Secondary 2 = 170 (52.15%) Tertiary = 131 (40.18%) NA = 8 (2.45%)	Only ages 18 + Secondary 1 = 15% Secondary 2 = 47% Tertiary = 38%
Income	*revenu {force response}* Finally, we need information on the net monthly income of your household. Think of your own income or pension, but also of the income or pension of any other persons in your household. Rest assured that this information will be treated strictly confidentially and anonymously	< 5001 CHF = 211 (34.82%) 5001–9000 CHF = 177 (29.21%) > 9000 CFH = 112 (18.48%) NA = 106 (17.49%)	< 5001 CH = 201 (34.96%) 5001–9000 CH = 189 (32.87%) > 9000 CH = 95 (16.52%) NA = 90 (15.65%)	< 5001 CH = 119 (36.50%) 5001–9000 CH = 93 (28.53%) > 9000 CH = 54 (16.56%) NA = 60 (18.4%)	< 5001 CH = 51.2% 5001–9000 CH = 37.7% > 9000 CH = 9.9%

**Table 5:** VAA items

German	English translation
Wir werden nun Ihre Meinung zu zehn Argumenten erfragen, welche in Zusammenhang mit der Abstimmung über das neue Energiegesetz (Energiestrategie 2050) stehen. Geben Sie jeweils an, wie stark Sie den Aussagen zustimmen	We are now going to ask your opinion about ten arguments related to the vote on the new energy law (*Energiestrategie 2050*). Please indicate how much you agree with each statement
Die öffentliche Unterstützung erneuerbarer Energien fördert Innovation und technologische Entwicklung	Public support for renewable energy sources fosters innovation and technological development
Es sollen keine neuen Kernkraftwerke gebaut werden	No new nuclear power plants should be built
Finanzielle Unterstützungen für energetische Gebäudesanierungen sind nötig	Financial support for energy-efficient renovations is necessary
Den Energieverbrauch zu senken ist notwendig und machbar	Decreasing energy consumption is necessary and feasible
Subventionen zur Förderung erneuerbarer Energien sind aktuell sinnvoll, sollen aber später durch ein Lenkungssystem abgelöst werden	Subsidies to foster renewable energy sources are currently sensible but should later be replaced by a steering system
Es ist nicht Aufgabe des Staates, die Energieproduktion zu steuern	It is not up to the government to regulate energy production
Erneuerbare Energien werden trotz staatlicher Investitionen die Energieversorgung nicht sicherstellen können	Despite government-funded renewable energy sources, energy supply cannot be guaranteed in future
Die mit der Energiestrategie 2050 verbundenen Regulierungen schaden der Wirtschaft	The regulations related to the new energy law (*Energiestrategie 2050*) are harming the economy
Durch die Förderung von Windenergie und Photovoltaik wird die Zerstörung natürlicher Lebensräume und die Zersiedelung vorangetrieben	The promotion of wind energy and solar energy expedites the destruction of natural habitats as well as urban sprawl
Es gibt keinen vom Menschen verursachten Klimawandel; deshalb sind die Massnahmen der Energiestrategie unnötig	There is no human-induced climate change; therefore, the measures of the energy strategy are unnecessary

*Note:* These ten items were presented to and evaluated by respondents in a randomized order. On each of these items, respondents first had to indicate their agreement or discontent with the item and then whether they deemed the item important or not. The figure below provides one example

The ten items were selected and formulated based on the arguments and conflicts present in the preceding parliamentary debate as well as in the referendum campaign. The ballot vote on the new energy law was framed and perceived not only as a vote on the specific law but more broadly on the Federal Council’s Energy Strategy 2050. For this reason, we also included several rather general items, e.g., whether respondents believed in human-induced climate change and whether the government should at all regulate energy production

## Footnotes

1 Our data do not allow us to differentiate between a persuasive and an intensifying *use* effect, so we only differentiate between the two in the context of the VAA’s message.

2 We refrain from formulating a corresponding hypothesis on the incongruence between party cues and the VAA message because we cannot test it empirically due to the small number of cases.

3 https://www.smartvote.ch/de/home.

4 Using 50, 100, and 200 as weighting factors is, of course, arbitrary to a certain extent. While the exact values do not matter as such (we could have also used it 0.5, 1, 2), their relative sizes do. A weight of 50 basically means that a statement is only half as important as a statement of average importance. As Figure S2 in the SI illustrates, however, the distribution of weights is rather similar across the different VAA items, and only very few respondents assigned low importance to any of the statements.

5 Unfortunately, an identical post-treatment question is not available for the control group.

6 https://www.bk.admin.ch/ch/d/pore/va/20170521/det612.html.

7 Table 4 in the Appendix suggests that the whole treatment group (including the early voters (n = 606) and the utilized subgroup of non-early voters (n = 326)) did not systematically differ from each other with respect to most sociodemographic variables and, importantly, political ideology and VAA outcome.

8 The results are identical when we use respondents’ original vote intentions from wave 2. When we use respondents’ vote intentions at the beginning of wave 3, their original vote intentions are significantly correlated with their final vote intentions, regardless of the direction of their votes, and the VAA outcome remains statistically significant (see Table S4 in the SI).

9 We further asked the respondents placed in the treatment group about their perceptions of the relevance of the VAA. Even though in most cases the VAA matched individuals’ original vote intentions, Figure S8 in the SI shows that almost 60 percent perceived the VAA as relevant, whereas only a quarter of the respondents indicated that they did not care about it. Furthermore, a majority of 58.3% wanted to consider the VAA when they formed their opinions. These findings corroborate the notion that the VAA can be considered relevant even if it documents a result that aligns with respondents’ own positions on the ballot proposal.

10 The low number of respondents who originally (wave 1) intended to vote “no” could prevent the interaction term from gaining statistical significance and could thus explain the lack of difference between original “no” voters and undecided respondents. However, this low number could not explain the similar marginal effects between the undecided respondents and those with an original intention to vote “no.”

11 We also considered a three-way interaction including original vote intention. However, the number of cases, especially that of original “no”-voters, is too small.

12 Similar to the coding of the vote intentions, we again opt for an asymmetric coding for the purpose of consistency but also because very few respondents received a very low VAA result.
