# Supplementary material for: The role of vote advice application in direct-democratic opinion formation: an experiment from Switzerland
Source: Acta Polit. 2022 Oct 26;58(4):792–818. doi: 10.1057/s41269-022-00264-5 (PMC11138372; doi:10.1057/s41269-022-00264-5)
Supplement: Supplementary file 1 — Supplementary file1 (DOCX 1020 kb) [file 41269_2022_264_MOESM1_ESM.docx]

# Supplemental material

**Overview**

**Section 1: Further descriptive analysis on vote intention and VAA result**

1. **Figure S1** VAA responses – print screen from original survey
2. **Figure S2** Distribution of argument weighting by respondents
3. **Figure S3** Distribution of VAA scores in the Treatment group
4. ***Table S1*** *Mobility in vote intentions at the individual level between waves*
5. **Figure S4** Comparison of control and treatment group
6. **Table S2** Voting intention control and treatment group
7. **Figure S5** Voting Intention before and after the treatment
8. **Figure S6** Scatterplot of voting intention and VAA score – pre- and post-treatment
9. ***Figure S7*** *Perception of the VAA result*
10. ***Figure S8*** *Perceived relevance of the VAA result*

**Section 2: Robustness checks of models**

1. ***Table S3*** *Full results of Table 2 (including control variables)*
2. **Table S4** Replication of Model 7 (Table 2) with different measurements of prior vote intention
3. **Table S5** Full Table 3 from the main text (including the control variables)
4. **Table S6** Replication of Model 9 (Table 3) with different measurements of prior vote intention

**Section 1: Further descriptive analysis on vote intention and VAA result**

**Figure S1** VAA responses – print screen from original survey

*
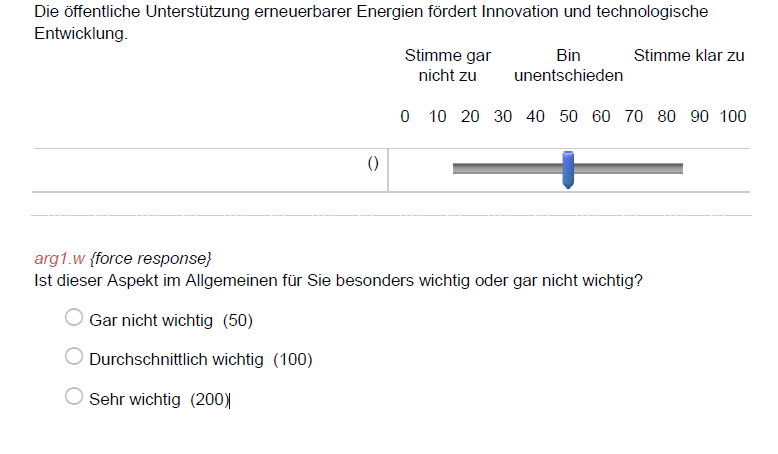
*

In general, is this aspect very important or not important at all to you?

o Not important at all (50)
o Of average importance (100)
o Very important (200)

**Figure S2** Distribution of argument weighting by respondents


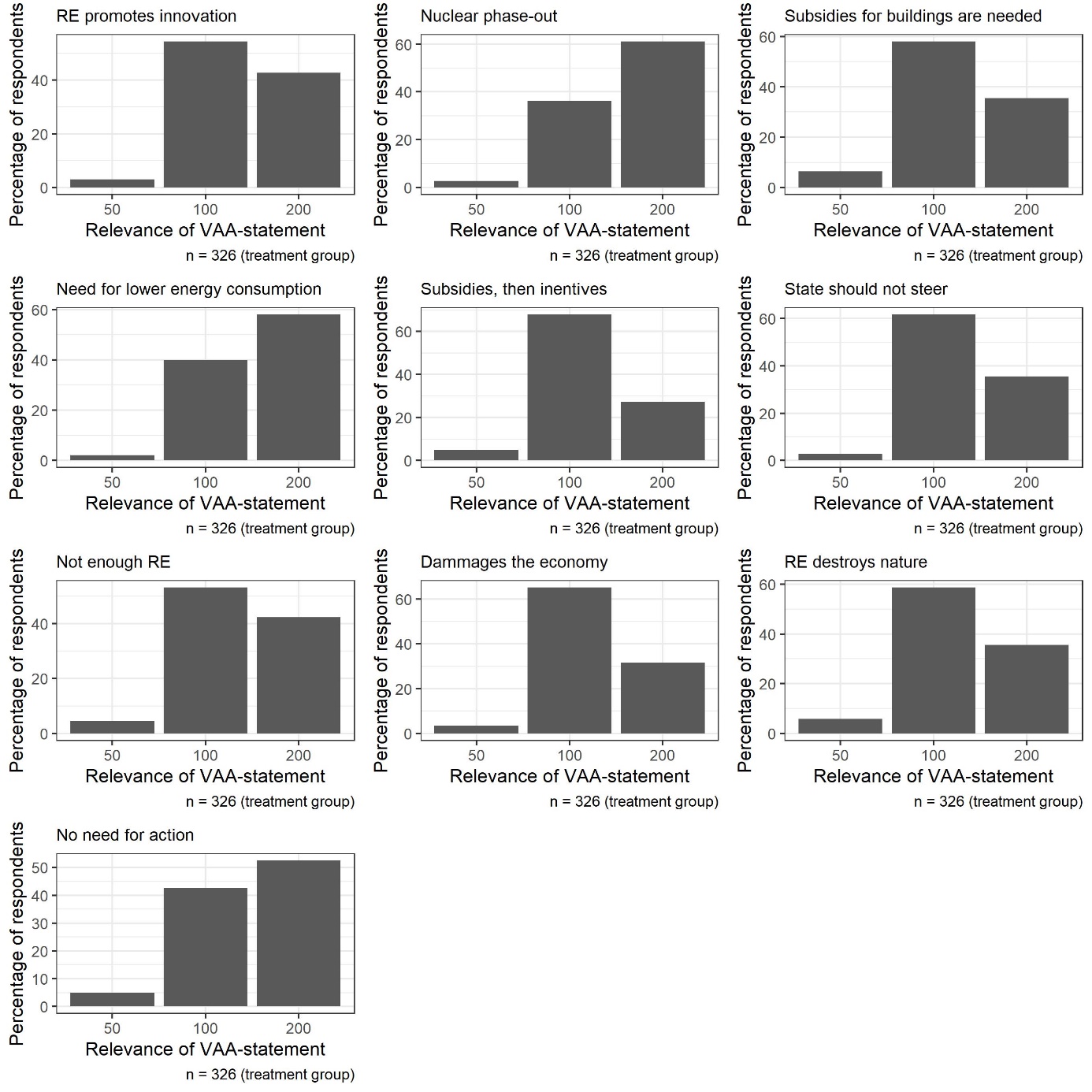


**Figure S3** Distribution of VAA scores in the Treatment group


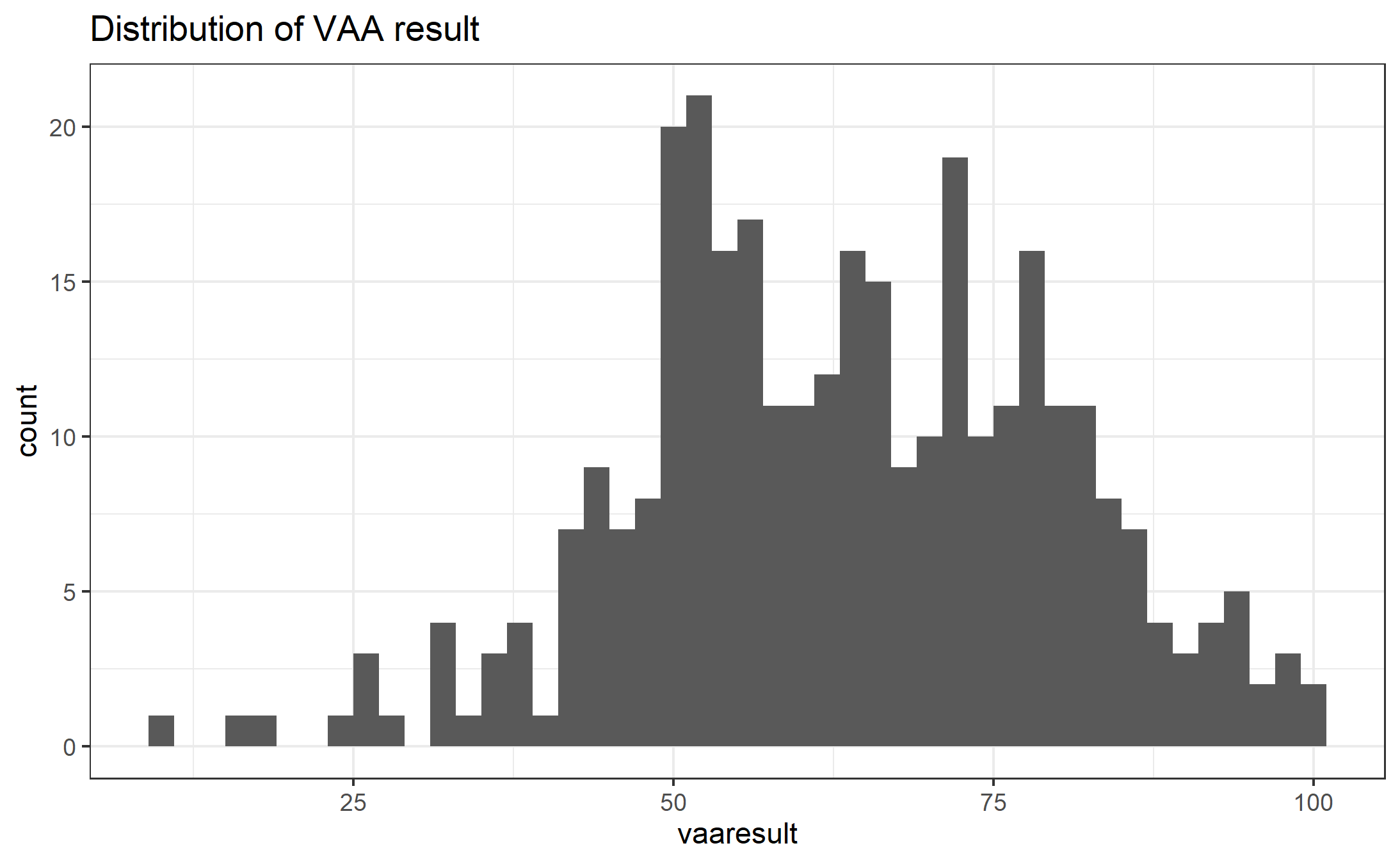


**Table S1** *Mobility in vote intentions at the individual level between waves*

|  | *W1: No* | W3 | | |
| --- | --- | --- | --- | --- |
|  |  | No | Undecided | Yes |
| W2 | No | ***56*** | 1 | 0 |
|  | Undecided | 5 | 4 | 3 |
|  | Yes | 1 | 0 | 3 |
|  |  |  |  |  |
|  | *W1: Undecided* | W3 | | |
|  |  | No | Undecided | Yes |
| W2 | No | 125 | 13 | 11 |
|  | Undecided | 96 | ***180*** | 137 |
|  | Yes | 18 | 27 | 183 |
|  |  |  |  |  |
|  | *W1: Yes* | W3 | | |
|  |  | No | Undecided | Yes |
| W2 | No | 21 | 5 | 5 |
|  | Undecided | 25 | 33 | 42 |
|  | Yes | 13 | 19 | ***155*** |

**Note:** Grey highlighted: Describes the group’s vote intention in wave 1. Columns depict the vote intention in wave 2, rows the vote intention in wave 3. The bold number denotes those with stable vote intention over the three waves. All others have at least changed vote intention between two waves.

**Figure S4** Comparison of control and treatment group


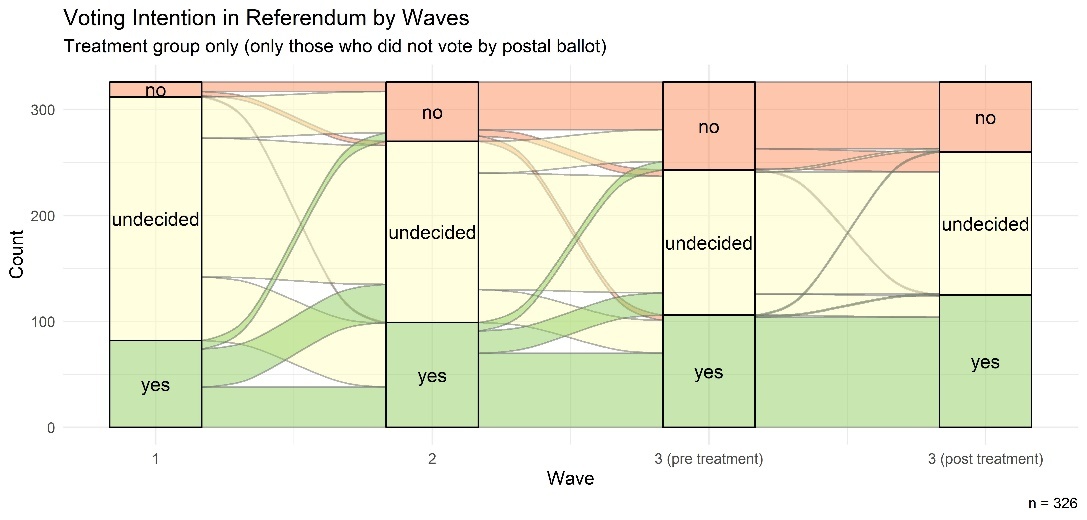


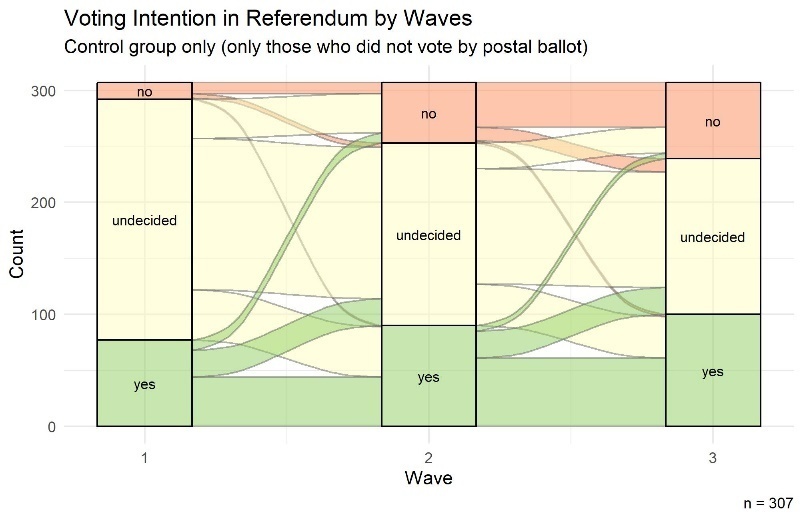


Vote intention across survey waves for both groups. Upper graph: Voters who received the VAA; Lower graph: Voters without VAA.

**Table S2** Voting intention control and treatment group

|  | **Control group, vote intention wave 3,**  **n = 307** | **Treatment group, vote intention wave 3 (pre-treatment), n = 326** | **Treatment group, vote intention wave 3 (post-treatment), n = 326** |
| --- | --- | --- | --- |
| No  Undecided  Yes | 0.22  0.45  0.33 | 0.25  0.42  0.33 | 0.20  0.41  0.38 |

*Note:* Share of respondents per category and group. Ordinal logistic regressions reveal that the differences between non-early voters in control group and treatment group (pre- and post-treatment) are not statistically significant.

**Figure S5** Voting Intention before and after the treatment (n = 326)


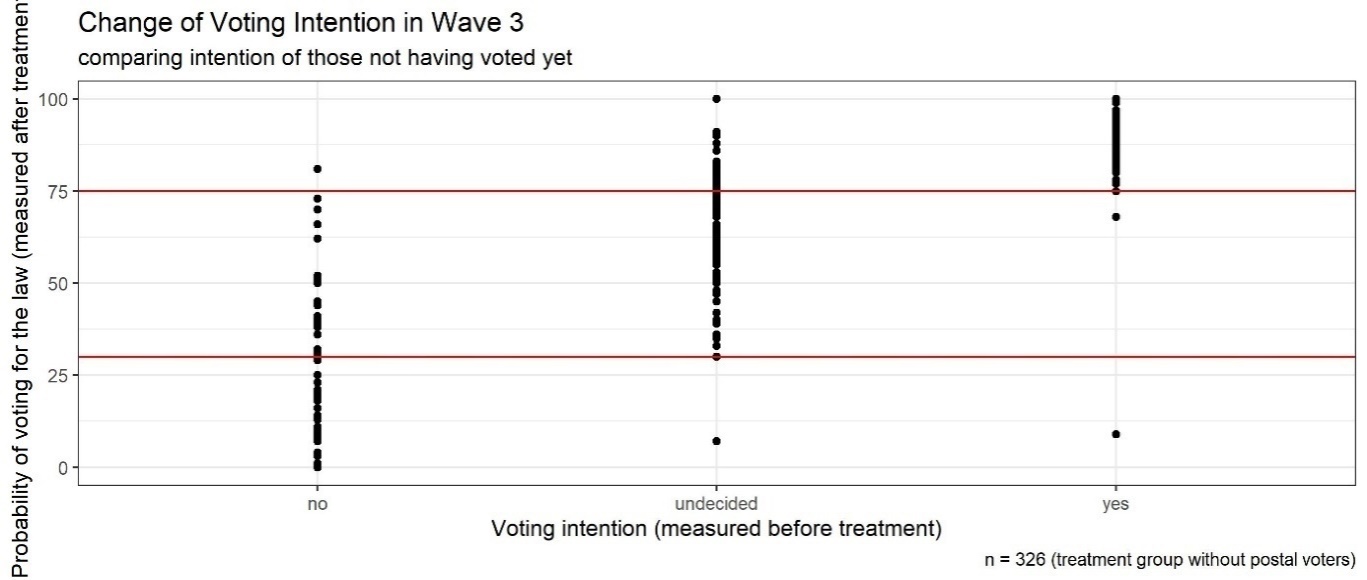


Note: Voting intention before the treatment has been measured categorically (“no”, “undecided”, “yes”); after the treatment, the probability of voting “yes” has been measured (0–100). Red lines have been added for comparison, indicating the cut-off points of a < 30 (probably voting no) and < 75 (probably voting yes) probability of voting yes. Reading example: Those who indicated voting yes before the treatment exhibited a very high likelihood (above 75%) of voting yes after the treatment. In the group with a prior “no” intention, some respondents indicated a likelihood of voting yes between 25 and 75%, i.e., they were less sure of voting no or even tended towards yes after having seen the VAA. Among the previously undecided group, a trend towards yes could be observed.

**Figure S6** Scatterplot of voting intention and VAA score – pre- and post-treatment


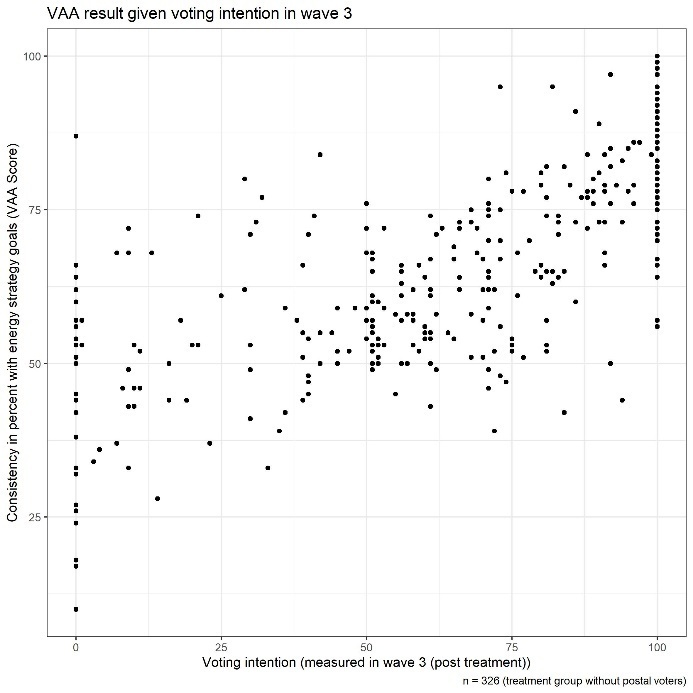
**
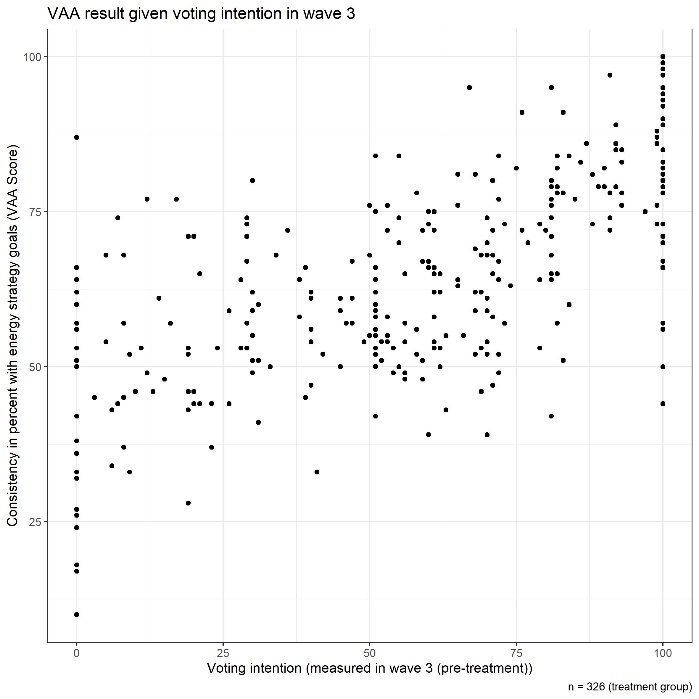
**

***Fig. S7*** *Perceptions of the VAA result*


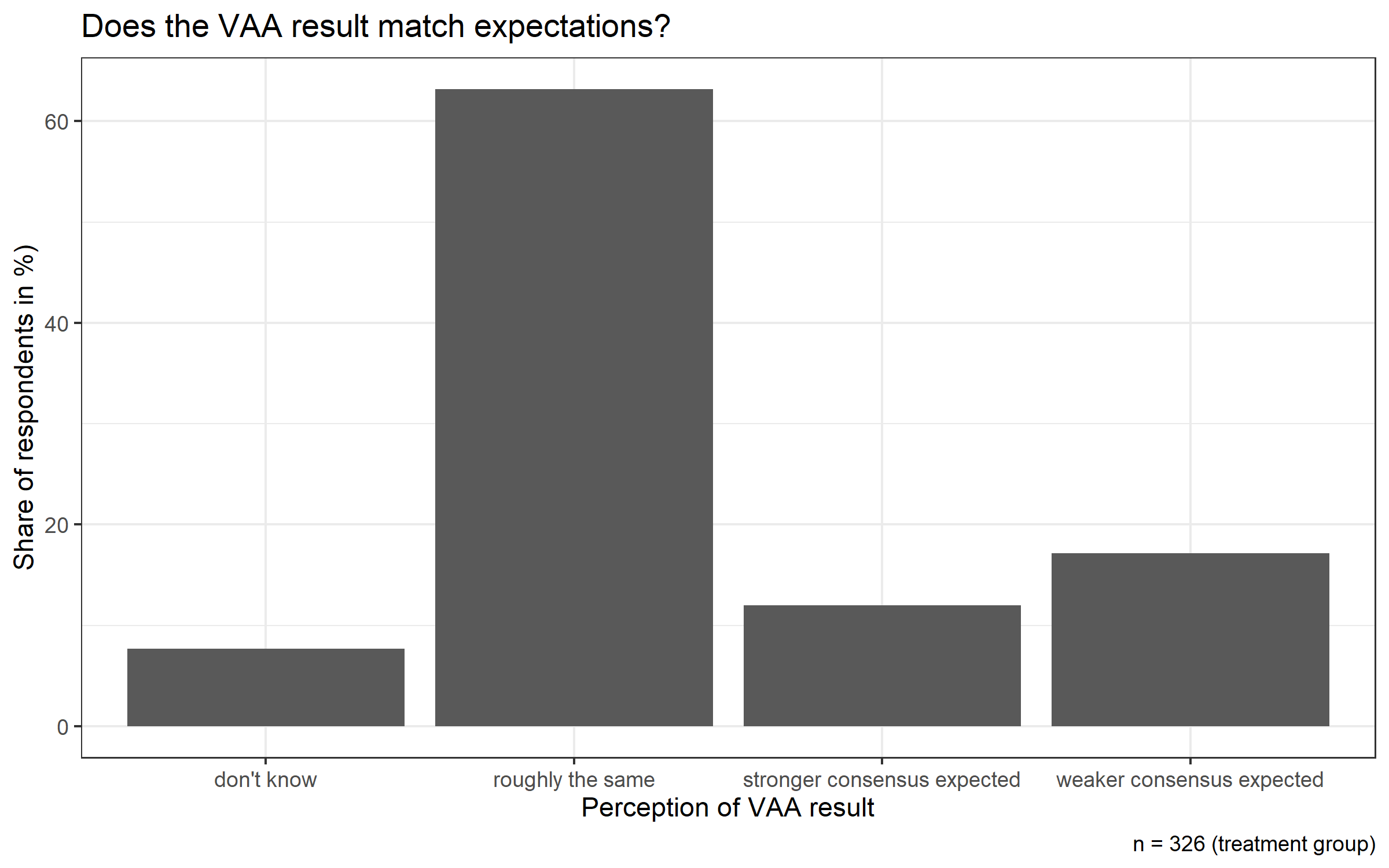


***Figure S8*** *Perceived relevance of the VAA result*


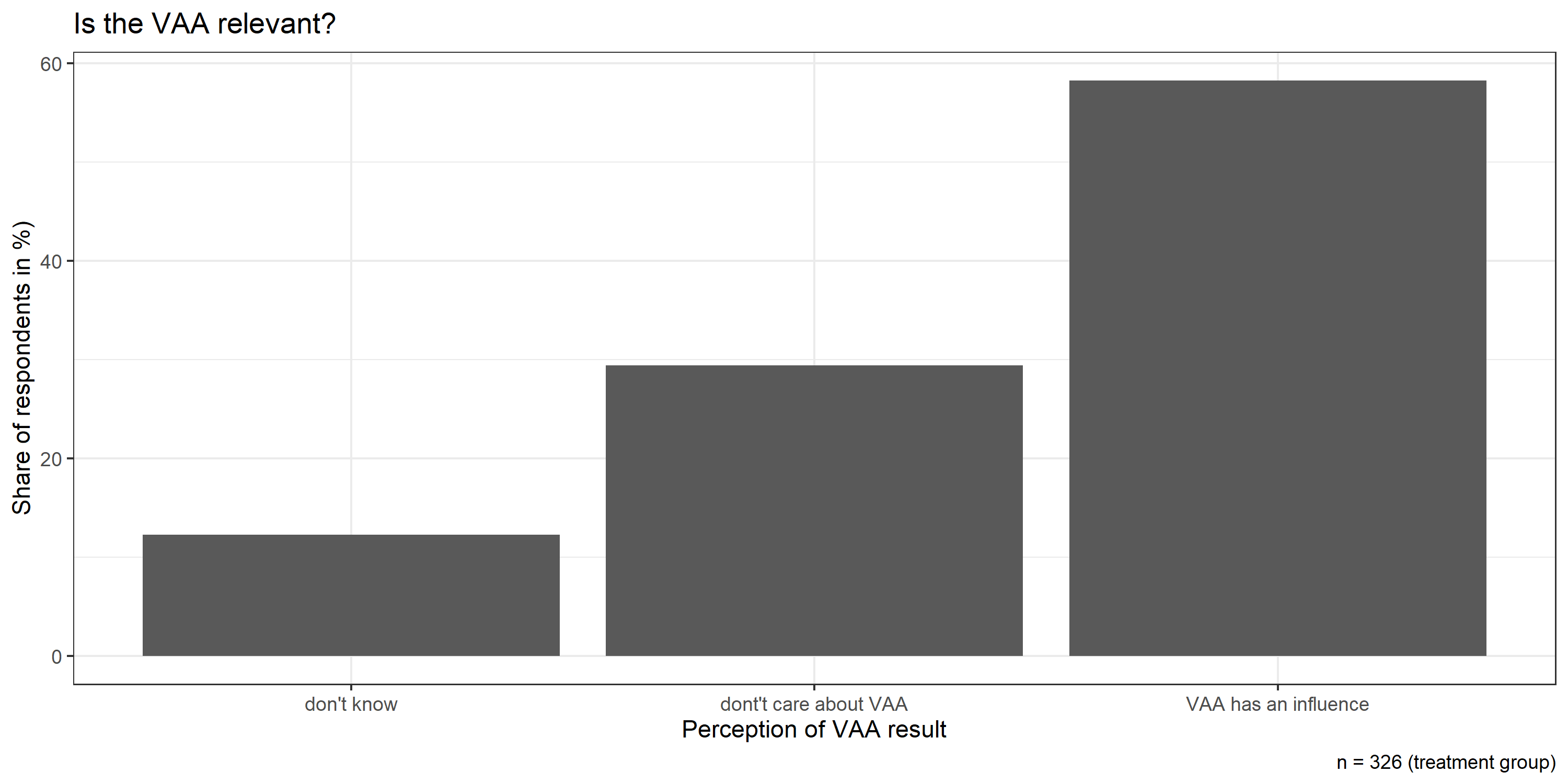


**Section 2: Further models**

**Table S3** *Full results of Table 2 (including control variables)*

|  | **(2)** | | **(2a)** | | **(3)** | | **(4)** | | **(5)** | | **(6)** | | **(7)** | |
| --- | --- | --- | --- | --- | --- | --- | --- | --- | --- | --- | --- | --- | --- | --- |
| *Predictors* | *Estimates* | *p* | *Estimates* | *p* | *Estimates* | *p* | *Estimates* | *p* | *Estimates* | *p* | *Estimates* | *p* | *Estimates* | *p* |
| Intercept | -27.65 | **<0.001** | 58.26 | **<0.001** | -28.88 | **<0.001** | -40.28 | **<0.001** | -37.6 | **<0.001** | -11 | 0.436 | -10.5 | 0.474 |
| VAA outcome (range 0 to 100) | 1.38 | **<0.001** |  |  | 1.37 | **<0.001** | 1.55 | **<0.001** | 1.47 | **<0.001** | 1.03 | **<0.001** | 1.02 | **<0.001** |
| Voting intention: no (ref = undecided) |  |  | -29.83 | **0.001** | 4.99 | 0.458 | 13.86 | 0.381 | 17.31 | 0.276 | 19.85 | 0.277 | 21.28 | 0.248 |
| yes |  |  | 13.57 | **0.001** | 6.5 | **0.031** | 51.32 | **<0.001** | 45.9 | **0.001** | 47.14 | **<0.001** | 41.25 | **0.002** |
| VAA outcome x voting intention no |  |  |  |  |  |  | -0.11 | 0.751 | -0.19 | 0.601 | -0.25 | 0.524 | -0.28 | 0.476 |
| VAA outcome x voting intention yes |  |  |  |  |  |  | -0.66 | **0.001** | -0.6 | **0.002** | -0.61 | **0.002** | -0.54 | **0.005** |
| Favorite energy party: left (ref = none) |  |  |  |  |  |  |  |  | 5.87 | 0.154 | -29.36 | 0.091 | -29.08 | 0.095 |
| centre |  |  |  |  |  |  |  |  | 6.03 | 0.15 | -33.6 | 0.06 | -33.14 | 0.067 |
| right |  |  |  |  |  |  |  |  | -6.07 | 0.195 | -38.54 | **0.036** | -41.74 | **0.023** |
| no answer |  |  |  |  |  |  |  |  | 1.39 | 0.736 | -15.89 | 0.378 | -17.98 | 0.319 |
| VAA outcome x left |  |  |  |  |  |  |  |  |  |  | 0.56 | **0.034** | 0.57 | **0.031** |
| VAA outcome x centre |  |  |  |  |  |  |  |  |  |  | 0.63 | **0.021** | 0.61 | **0.028** |
| VAA outcome x right |  |  |  |  |  |  |  |  |  |  | 0.54 | 0.073 | 0.61 | **0.042** |
| VAA outcome x no answer |  |  |  |  |  |  |  |  |  |  | 0.29 | 0.308 | 0.3 | 0.286 |
| Age: 18-24 (ref = 25-44) |  |  |  |  |  |  |  |  |  |  |  |  | 6.38 | 0.191 |
| 45-64 |  |  |  |  |  |  |  |  |  |  |  |  | -8.03 | **0.006** |
| 65+ |  |  |  |  |  |  |  |  |  |  |  |  | -2.17 | 0.584 |
| Sex: female (ref = male) |  |  |  |  |  |  |  |  |  |  |  |  | -2.12 | 0.426 |
| Household income: 5001-9000 (ref = <5000) |  |  |  |  |  |  |  |  |  |  |  |  | 4.29 | 0.184 |
| >9000 |  |  |  |  |  |  |  |  |  |  |  |  | 5.72 | 0.144 |
| no answer |  |  |  |  |  |  |  |  |  |  |  |  | 6.07 | 0.105 |
| Education: Secondary I (ref = Secondary II) |  |  |  |  |  |  |  |  |  |  |  |  | -0.07 | 0.99 |
| Tertiary |  |  |  |  |  |  |  |  |  |  |  |  | 1.74 | 0.523 |
| no answer |  |  |  |  |  |  |  |  |  |  |  |  | 14.47 | 0.084 |
| Observations | 326 | | 326 | | 326 | | 326 | | 326 | | 326 | | 326 | |
| R^2^ / R^2^ adjusted | 0.486 / 0.485 | | 0.077 / 0.071 | | 0.494 / 0.489 | | 0.513 / 0.505 | | 0.527 / 0.514 | | 0.538 / 0.519 | | 0.566 / 0.533 | |

**Table S4** Replication of Model 7 (Table 2) with different measurements of prior vote intention

|  | **(7.2)** | | **(7.3)** | |
| --- | --- | --- | --- | --- |
| *Predictors* | *Estimates* | *p* | *Estimates* | *p* |
| Intercept | 19.15 | 0.167 | 34.09 | **0.001** |
| VAA outcome (range 0 to 100) | 0.61 | **0.005** | 0.42 | **0.012** |
| Voting intention*: no (ref = undecided) | -17.52 | 0.158 | -34.52 | **<0.001** |
| yes | 29.15 | **0.037** | 40.90 | **0.001** |
| Favorite energy party: left (ref = none) | -26.08 | 0.106 | -1.95 | 0.862 |
| centre | -44.28 | **0.007** | -17.99 | 0.116 |
| right | -34.54 | **0.034** | -18.90 | 0.109 |
| no answer | -34.12 | **0.034** | -12.84 | 0.257 |
| Age: 18–24 (ref = 25–44) | 7.34 | 0.099 | 2.26 | 0.471 |
| 45–64 | -6.19 | **0.022** | -1.40 | 0.461 |
| 65+ | -1.08 | 0.762 | 4.76 | 0.060 |
| Sex: female (ref = male) | -1.76 | 0.469 | 0.47 | 0.782 |
| Household income: 5001–9000 (ref = <5000) | 3.98 | 0.174 | 0.95 | 0.645 |
| >9000 | 1.53 | 0.670 | -0.32 | 0.899 |
| no answer | 3.68 | 0.271 | 1.73 | 0.463 |
| Education: Secondary I (ref = Secondary II) | 1.54 | 0.770 | -3.79 | 0.312 |
| Tertiary | 2.80 | 0.253 | -0.57 | 0.744 |
| no answer | 10.87 | 0.152 | 10.09 | 0.058 |
| VAA outcome x voting intention* no | -0.07 | 0.724 | -0.06 | 0.715 |
| VAA outcome x voting intention* yes | -0.24 | 0.221 | -0.27 | 0.110 |
| VAA outcome x left | 0.49 | **0.045** | 0.13 | 0.442 |
| VAA outcome x centre | 0.74 | **0.003** | 0.30 | 0.088 |
| VAA outcome x right | 0.46 | 0.082 | 0.32 | 0.092 |
| VAA outcome x no answer | 0.51 | **0.044** | 0.18 | 0.316 |
| Observations | 326 | | 326 | |
| R^2^ / R^2^ adjusted | 0.642 / 0.615 | | 0.822 / 0.809 | |

*Note:* Prior vote intention measured in wave 2 (7.2) and wave 3 (7.3).

**Table S5** Full Table 3 from the main text (including the control variables)

|  | **(8)** | | **(9)** | | |  |
| --- | --- | --- | --- | --- | --- | --- |
| *Predictors* | *Estimates* | *p* | | *Estimates* | *p* | |
| Intercept | 48.22 | **<0.001** | | 44.52 | **<0.001** | |
| Voting intention (w1) vs. VAA (ref = undecided & undecided)  no & no | -48.22 | **<0.001** | | -46.41 | **<0.001** | |
| no & undecided | -9.85 | 0.287 | | -7.72 | 0.408 | |
| no & yes | 42.78 | 0.095 | | 40.4 | 0.116 | |
| undecided & no | -43.55 | **0.004** | | -45.96 | **0.002** | |
| undecided & yes | 41.36 | **<0.001** | | 41.58 | **<0.001** | |
| yes & undecided | 12.82 | **0.002** | | 11.88 | **0.004** | |
| yes & yes | 41.36 | **<0.001** | | 41.62 | **<0.001** | |
| Age (ref = 25–44)  18–24 |  |  | | 7.85 | 0.155 | |
| 45–64 |  |  | | -7.09 | **0.032** | |
| 65+ |  |  | | -2.27 | 0.606 | |
| Sex: female (ref = male) |  |  | | 0.13 | 0.964 | |
| Household income (ref = <5000 CHF)  5001–9000 CHF |  |  | | 5.75 | 0.113 | |
| >9000 CHF |  |  | | 5.94 | 0.181 | |
| no answer |  |  | | 6.16 | 0.143 | |
| Education (ref = Secondary II)  Secondary I |  |  | | -0.7 | 0.916 | |
| Tertiary |  |  | | 3.89 | 0.207 | |
| no answer |  |  | | 15.39 | 0.098 | |
| Observations | 326 | | 326 | | |  |
| R^2^ / R^2^ adjusted | 0.393 / 0.380 | | 0.426 / 0.394 | | |  |

**Table S6** Replication of Model 9 (Table 3) with different measurements of prior vote intention

|  | **(9.2)** | | **(9.3)** | |
| --- | --- | --- | --- | --- |
| *Predictors* | *Estimates* | *p* | *Estimates* | *p* |
| Intercept | 50.88 | **<0.001** | 57.45 | **<0.001** |
| Voting intention* vs. VAA: no & no (ref = undecided & undecided) | -50.83 | **<0.001** | -56.77 | **<0.001** |
| no & undecided | -33.56 | **<0.001** | -40.86 | **<0.001** |
| no & yes | 42.03 | **<0.001** | -23.87 | **0.002** |
| undecided & yes | 26.63 | **<0.001** | 15.86 | **<0.001** |
| yes & undecided | 18.50 | **<0.001** | 27.47 | **<0.001** |
| yes & yes | 40.52 | **<0.001** | 36.36 | **<0.001** |
| Age: 18–24 (ref = 25–44) | 7.06 | 0.131 | 2.46 | 0.453 |
| 45–64 | -5.85 | **0.037** | -0.35 | 0.858 |
| 65+ | -3.54 | 0.339 | 4.72 | 0.074 |
| Sex: female (ref = male) | -0.39 | 0.878 | 1.98 | 0.263 |
| Household income: 5001–9000 (ref = <5000) | 5.68 | 0.063 | 1.31 | 0.543 |
| >9000 | 0.79 | 0.831 | -1.27 | 0.630 |
| no answer | 4.80 | 0.166 | 0.65 | 0.790 |
| Education: Secondary I (ref = Secondary II) | 0.36 | 0.948 | -5.10 | 0.192 |
| Tertiary | 4.70 | 0.068 | -0.37 | 0.838 |
| no answer | 9.29 | 0.236 | 8.41 | 0.126 |
| Observations | 326 | | 326 | |
| R^2^ / R^2^ adjusted | 0.592 / 0.571 | | 0.797 / 0.787 | |

*Note:* Prior vote intention measured in wave 2 (9.2) and wave 3 (9.3). Based on waves 2 and 3, no observations exist in the category “vote intention undecided” & VAA outcome “no”.
